# Meat-Borne-Parasite: A Nanopore-Based Meta-Barcoding Work-Flow for Parasitic Microbiodiversity Assessment in the Wild Fauna of French Guiana

**DOI:** 10.3390/cimb46050237

**Published:** 2024-04-24

**Authors:** Adria Matoute, Simone Maestri, Mona Saout, Laure Laghoe, Stéphane Simon, Hélène Blanquart, Miguel Angel Hernandez Martinez, Magalie Pierre Demar

**Affiliations:** 1 Tropical Biome and Immunopathophysiology (TBIP), Université de Guyane, 97300 Cayenne, France; adria.matoute@univ-guyane.fr (A.M.); simone.maestri@hotmail.it (S.M.); mona.saout@univ-guyane.fr (M.S.); laure.laghoe@gmail.com (L.L.); stef240572@gmail.com (S.S.); 2 U1019-UMR 9017-CIIL-Center for Infection and Immunity of Lille, Institut Pasteur de Lille, CHU Lille, INSERM, CNRS, Université Lille, 59000 Lille, France; 3 GenoScreen, 5900 Lille, France; 4 Laboratoire Associé du CNR Leishmaniose, Laboratoire Hospitalo-Universitaire de Parasitologie et Mycologie, Centre Hospitalier Andrée Rosemon, 97300 Cayenne, France; miangher@homail.com

**Keywords:** Apicomplexa, Amazon, NGS sequencing, meta-barcoding, wild mammals

## Abstract

French Guiana, located in the Guiana Shield, is a natural reservoir for many zoonotic pathogens that are of considerable medical or veterinary importance. Until now, there has been limited data available on the description of parasites circulating in this area, especially on protozoan belonging to the phylum Apicomplexa; conversely, the neighbouring countries describe a high parasitic prevalence in animals and humans. Epidemiological surveillance is necessary, as new potentially virulent strains may emerge from these forest ecosystems, such as Amazonian toxoplasmosis. However, there is no standard tool for detecting protozoa in wildlife. In this study, we developed Meat-Borne-Parasite, a high-throughput meta-barcoding workflow for detecting Apicomplexa based on the Oxford Nanopore Technologies sequencing platform using the 18S gene of 14 Apicomplexa positive samples collected in French Guiana. Sequencing reads were then analysed with MetONTIIME pipeline. Thanks to a scoring rule, we were able to classify 10 samples out of 14 as Apicomplexa positive and reveal the presence of co-carriages. The same samples were also sequenced with the Illumina platform for validation purposes. For samples identified as Apicomplexa positive by both platforms, a strong positive correlation at up to the genus level was reported. Overall, the presented workflow represents a reliable method for Apicomplexa detection, which may pave the way for more comprehensive biomonitoring of zoonotic pathogens.

## 1. Introduction

The Amazon biome stretches to the extensive Amazon basin, a territory delimited by the Andean regions in the west and the Cerrado in the south. It covers part of Brazil (49%), Bolivia (11%), Peru (16%), Ecuador, Colombia, Venezuela, Guyana, Suriname, and French Guiana and hosts a wide range of wild fauna of different ecosystems. Mammals are a potential reservoir for many zoonotic pathogens, including Apicomplexa taxa such as *Sarcocystis* spp., *Cryptosporidium* spp., *or Toxoplasma gondii* [1,2]. This phylum is a diverse group of protozoan parasites that are unicellular eukaryotes and obligate intracellularly without flagella, and they were detected in different mammalian organs [3,4,5]. Using their apical complex and their secretory organelle structure, they invade the host cell [6], potentially causing chronic asymptomatic diseases or severe acute diseases [7].

In South America, many countries describe a high prevalence of these parasites in animal meat, as *Sarocystis* spp. is in the tongue muscles of armadillos [8], in wild birds [9] and *Toxoplasma gondii* and *Sarcocystis* spp. are detected in the *Alouatta guariba clamitans* [5]. Except for *T. gondii* [3,10], there are no data on the description of such parasites circulating in French Guiana, strongly hampering the assessment of the risk of human transmission. Indeed, humans can be infected through activities such as hunting, fishing in or consumption of soiled water, raw or uncooked game meat [11,12,13], which are greatly enjoyed by the population. Environmental changes induced by deforestation and urbanization enable close exchanges between different ecosystems, causing a disruption in the sylvatic cycle. The anthropization of natural environments exposes human population to the risks of the emergence of new virulent strains from wildlife. There is, therefore, a risk of the introduction, circulation, and emergence of new parasitic species, which are usually not adapted to humans, potentially causing severe pathologies, as described for Amazonian toxoplasmosis [14].

Currently, there is no standard tool for the detection of protozoa in wild animals. Indeed, in the few studies about protozoa in meat, the authors perform conventional PCR using 18S rRNA gene general primers, or also target Apicomplexa with specific primers to amplify one parasitic species of the phylum [5,15,16]. Others also employed the Indirect Fluorescent Antibody Test (IFAT), which relies on detecting antibodies against Apicomplexa, and is known for its high risk of cross reactions [17]. All these conventional serological or molecular methods are not suitable for the detection of co-infections. The development of high-throughput sequencing technologies is an approach now widely used in the environmental field to assess the diversity of microorganisms [18,19]. High-throughput sequencing platforms allow us to comprehensively study and better reveal the diversity of the species and co-infections present in a sample [20].

In this study, we developed a low-cost, portable, and fast workflow based on Nanopore sequencing to target the 18S rRNA gene for Apicomplexa detection. The Nanopore reads were analysed using the MetONTIIME pipeline, which fosters reproducibility and standardization, through the underlying QIIME2 [21] and Nextflow [22] frameworks. Moreover, thanks to the sequencing of matched samples on the Illumina platform, we validated the workflow and showed high reliability of taxa abundances at the genus level.

## 2. Materials and Methods

### 2.1. Sampling and Samples

The animals studied were from the TBIP collection, which has been set up during the PARALIM project, whose aim was to assess the dietary risks associated with the consumption of wild animals; this is commonly practiced in French Guiana. Therefore, it was possible to identify the potential food risks but also the ecological risks threatening the survival of animal species. It is with this interest that a massive collection of animal organs from French Guiana was built. This collection of the TBIP laboratory was constituted on a voluntary basis and without financial compensation from hunters, slaughterhouses, and veterinarians between 2015 and 2019. The animal species were identified using the Ivanova et al. protocol [23].

For this study, the samples were selected according to (i) their geographical origin: from the Cayenne Island region (from Cayenne, Dégrad-des-Cannes, Matoury, Larivot and Stoupan), the eastern region (Cacao, Saint-Georges, National Road 2 and Bélizon), the western region (from Sinnamary to Grand-Santi), the savannahs (from Montsinery to Kourou); (ii) the type of organ selected according to knowledge of parasite cycles including those consumed by the local population; and (iii) the species of the animals collected, and the lifestyle and diet of the species that may influence the risk of their exposure to parasites.

Then, we selected 20 samples positive for Apicomplexa, according to a PCR test, for the development of Meat-Borne-Parasite, a meta-barcoding workflow based on the Oxford Nanopore Technologies sequencing platform.

### 2.2. Molecular Analysis

#### 2.2.1. DNA Extraction

DNA purification was carried out using the QIAmp DNA mini kit (Qiagen, Paris, France) according to the supplier’s recommendations. Lysis was performed overnight by adding 180 µL of the ATL lysis buffer and 20 µL of proteinase K to the tissue sample (<25 mg). Then, DNA was extracted from the lysate and the eluate was collected in 200 µL of elution buffer. A negative extraction control was included in the set by replacing the biopsy with water.

#### 2.2.2. DNA Amplification

Detection of apicomplexan parasites was performed using a conventional PCR with the 800 bp DNA fragment encoding the universal eukaryote 18S ribosomal RNA (18S rRNA) gene [24]. A set of primers ApiF18Sv1v5 (5′-GCC ATG CAT GTC TAA GTA TAA GCT TT-3′) and ApiR18Sv1v5 (5′-CTT TAA CAA ATC TAA GAA TTT CACC TCT G-3′) targeting V1 to V5 regions of the 18S rRNA gene were designed by the TBIP laboratory.

The mixture reaction was run in a final volume of 50 µL containing 10 µL of Hot Fire Polymerase (HFP) enzyme (Solis Biodyne, Tartu, Estonia), 2 µL of primer sense and antisense (5 µM), 33 µL of water, and 5 µL of the DNA sample. Using the Arktik thermocycler^®^ (Thermo Fisher Scientific, Waltham, MA, USA), the following PCR conditions were used: an initial denaturation at 95 °C for 15 min, followed by 40 cycles of denaturation at 95 °C for 30 s, annealing at 54 °C for 45 s, and an extension at 72 °C for 90 s. The last stage was final elongation at 72 °C for 5 min. A positive and a negative control containing known genomic DNA and water, respectively, were included. The amplification products were visualized in a transilluminator after migration of the samples using electrophoresis in 1.2% agarose TBE gel. These 20 positive PCR amplicons were purified in solution according to the FastGene kit (NIPPON Genetics EUROPE, Düren, Germany), and were then quantified using the Qubit^®^ (Qiagen, Paris, France).

#### 2.2.3. Nanopore Library Preparation and Sequencing

A Nanopore sequencing library was built following the ligation sequencing kit and native barcoding kit (SQK-LSK109 with EXP-NBD196) protocol (Oxford Nanopore Technologies, London, UK) according to the manufacturer’s instructions. The library was then loaded on a R9.4.1 flow-cell^®^ (FLO-MIN106D, Oxford Nanopore Technologies, London, UK), and sequencing was carried out on a MinION^®^ Nanopore sequencer (Oxford Nanopore Technologies, London, UK) for 8 h using MinKNOW v22.10.7.

#### 2.2.4. Illumina Library Preparation and Sequencing

DNA extraction was performed from genomic DNA of matched samples. Then, the PCR amplification was carried out using the primer pair P1-TAReuk454FWd1-18S (5′-CCA GCA SCY GCG GTA ATT CC-3′) and P1-TAReuk454REV3-18S (5′-ACT TTC GTT CTT GAT YRA-3′), targeting the V4 region of the 18S rRNA gene [25]. A Diatomea strain was included as a positive control together with two negative controls, which were, respectively, the tissue extraction control and the PCR background of the total library preparation process.

Sequencing of the amplicon libraries was performed in a single Illumina MiSeq^®^ paired-end run with 2 × 250 bp read chemistry according to the Metabiote^®^ protocol for 18S gene sequencing (GenoScreen, Lille, France).

### 2.3. Bioinformatics Processing

#### 2.3.1. Meta-Barcoding Pipeline for Nanopore Data Analysis

Nanopore reads were base-called using Guppy v6.3.9 integrated into MinKNOW v22.10.7 with the “hac” model, and demultiplexing was performed requiring the presence of barcodes at both ends. Reads were then analysed with a novel bioinformatic pipeline, called MetONTIIME, based on the Nextflow workflow manager and QIIME2 environment [21,22]. In particular, reads with quality > 7 were filtered with NanoFilt [26] and compressed to the fastq.gz format. Reads were then imported in qiime2 v.2022.8.0 using “qiime tools import” and dereplicated using “qiime vsearch dereplicate-sequences” and “qiime vsearch cluster-features-de-novo”, to obtain a set of representative sequences and the corresponding table with read counts. Representative sequences were then aligned to the Silva_132_99_18S database (accessed on 5 January 2023) using “qiime feature-classifier classify-consensus-vsearch” requiring a minimum alignment identity of 90%, a minimum query coverage of 80%, and performing consensus taxonomy assignment among the top three hits. Taxonomy tables were then filtered, retaining only taxa belonging to Apicomplexa phylum. A scoring rule was developed for classifying a sample as Apicomplexa positive in case the number of reads of the sample assigned to Apicomplexa represents at least a 5-fold increase compared to the average number of reads from negative controls assigned to Apicomplexa in the same sequencing run. Accordingly, samples with less than 10 reads assigned to Apicomplexa were classified as Apicomplexa negative and were dropped from the analysis. Taxonomy tables and barplots describing the taxonomic classification at each taxonomic level were generated with “qiime taxa collapse” and “qiime taxa barplot”. All scripts for running the MetONTIIME pipeline are reported in https://github.com/MaestSi/MetONTIIME repository (accessed on 5 January 2023).

#### 2.3.2. Meta-Barcoding Pipeline for Illumina Data Analysis

Reads were analysed with the QIIME2_Illumina pipeline. In particular, fastq.gz files were imported in qiime2 v2022.8.0 using “qiime tools import” after generation of manifest.txt file, and PCR primers were trimmed with “qiime cutadapt trim-paired”. Overlapping mates were then merged with “qiime dada2 denoise-paired”. A set of amplicon sequence variants (ASVs) was obtained, together with a feature table, describing the occurrence of ASVs in each sample. The database Silva_132_99_18S (accessed on 5 January 2023) was then imported with “qiime tools import”, and then “qiime feature- classifier extract-reads” and “qiime feature-classifier fit-classifier-naive-bayes” were used to train a naïve Bayes classifier on the region of the 18S gene amplified using TAReuk454FWD1-TAReukREV3 primers. ASVs were then classified with “qiime feature-classifier classify-sklearn”. Taxonomy tables were then filtered retaining only taxa belonging to Apicomplexa phylum, and samples with no reads assigned to Apicomplexa were dropped from the analysis. Taxonomy tables and barplots were generated with “qiime taxa collapse” and “qiime taxa barplot”. All scripts for running the pipeline are reported in the https://github.com/MaestSi/QIIME2_Illumina repository (accessed on 5 January 2023).

#### 2.3.3. Comparison of Nanopore and Illumina Meta-Barcoding Results

Bioinformatic analysis was carried out to obtain taxonomic assignments for both platforms using Silva [27] as a reference database (accessed on 5 January 2023). Feature tables reporting the relative frequencies of taxa for Apicomplexa positive samples, obtained for both Nanopore and Illumina platforms, were merged into a single feature table. Relative frequencies for all the taxa collapsed at different taxonomic levels were then compared between the two platforms, and scatterplots were produced with ggplot2 [28]. The Pearson correlation was then computed between relative frequencies obtained with the two platforms, and a t-test was performed to estimate the probability of the association being null.

## 3. Results

### 3.1. Sampling and Apicomplexa Positive Samples Identification

**Figure 1:**
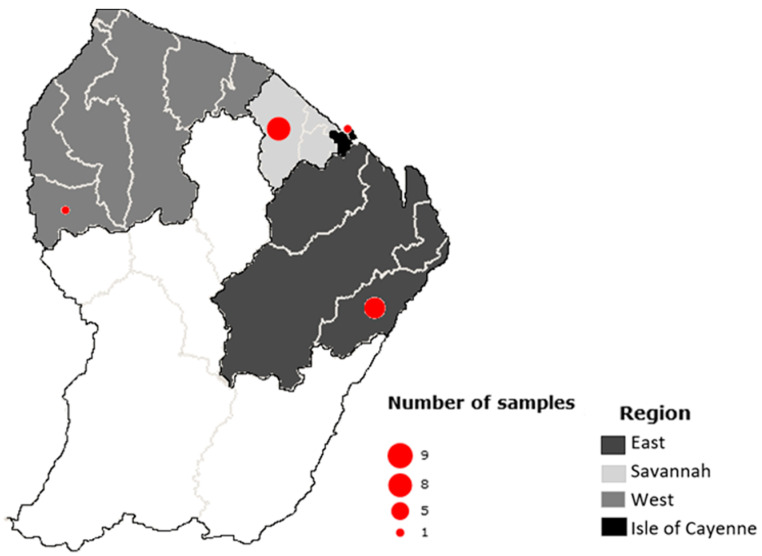
Geographical origin distribution of the individuals analysed.

In order to set-up and validate the Meat-Borne-Parasite workflow, a total of 20 samples from 15 individuals were first included in the study. These samples were from six different animal species (Table 1) and were obtained from lung (5), heart (7), tongue (7), and brain (1) tissues. They were collected in four main regions of the country: one in the Cayenne Island region, seven in the eastern region, one in the western region, and nine in the savannahs (Figure 1). Only 14 of them showed a clear amplicon in the gel; the remaining samples were, therefore, dropped and excluded from the following analyses.

### 3.2. Matched Samples Sequencing with Nanopore and Illumina Platforms

The Apicomplexa positive samples were then processed and sequenced in parallel with Nanopore and Illumina platforms. In particular, samples for Nanopore sequencing were PCR amplified with ApiF18Sv1v5 and ApiR18Sv1v5 primers, and sequencing was carried out for 8 h on a MinION device, producing 233,676 reads in total (Table 2), while samples for Illumina sequencing were PCR amplified with P1-TAReuk454FWd1-18S and P1-TAReuk454REV3-18S primers, and sequencing was carried out on a MiSeq instrument with a 2 × 250 paired-end mode, producing 1,569,910 reads in total (Table 2).

### 3.3. Taxonomy Assignment and Platforms Comparison

Bioinformatic analysis of sequencing reads was carried out with MetONTIIME and QIIME2_Illumina pipelines, respectively, to obtain taxonomic assignment. The negative controls showed no reads assigned to Apicomplexa for the Illumina platform, while up to two reads from the negative controls were assigned to Apicomplexa for the Nanopore platform, possibly due to demultiplexing errors. Reads from positive controls were assigned to the expected taxa.

**Figure 2:**
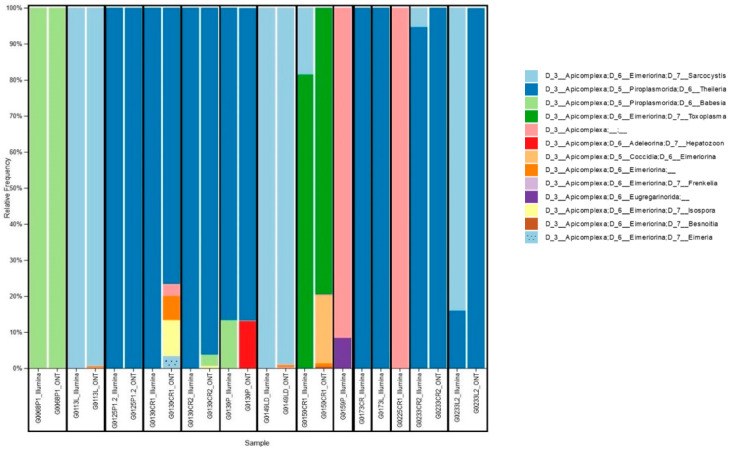
Apicomplexa relative frequency in wildlife samples. For each sample, the relative frequency of reads assigned to Apicomplexa for Illumina and Nanopore matched samples at level 6 (i.e., up to genus) is reported. G0***L1 indicates the host ID, the first four elements indicate its code, followed by the letter representing the organ studied (P: lung; L or LD: tongue; CR: heart; R: spleen), and then the number representing the organ number.

Only reads assigned to Apicomplexa were retained for further analyses (Figure 2). Nanopore platform classified 10 out of 14 samples as Apicomplexa positive, according to a scoring rule we developed, which classifies a sample as Apicomplexa positive in case the number of reads assigned to Apicomplexa is at least 5-fold the average number of reads assigned to Apicomplexa for negative controls. This scoring rule was adapted from previous works describing the adoption of Nanopore sequencing for pathogen detection [29,30], while the Illumina platform classified all 14 samples as Apicomplexa positive. In particular, the four samples classified as Apicomplexa negative by the Nanopore platform had a very low percentage of reads assigned to Apicomplexa also in the Illumina analysis, namely 0.22%, 0.09%, 0.04%, and 0.02% for the G0159P, G0173CR, G0173L, and G0225CR1 samples, respectively. The host species corresponding to the identification codes are reported in Table 3.

Interestingly, we reported some cases of co-carriage. For example, in sample G0130CR2, we detected only *Theileria* spp. with Illumina, while Nanopore detected *Theileria* spp., *Babesia* spp., and *Isospora* spp. Conversely, in sample G0233CR2, Nanopore detected only *Theileiria* spp., while Illumina detected both *Theileria* spp. and *Sarcocystis* spp.

Co-carriers were found in 28.5% (4/14) of samples studied with Illumina sequencing and 90% (9/10) of samples studied with Nanopore technology. Among the organs studied, we found certain parasites in a single organ, such as *T. gondii* in the heart. On the other hand, parasites such as *Babesia* spp. and *Theileria* spp. were found in all the organs analysed: heart, lung, and tongue.

Four protozoan families were identified through sequencing analysis: *Piroplasmorida*, *Eimeriorina*, *Coccidia*, *Adeleorina*, and *Eugregarinorida*. Among these families, four main genera (*Sarcocystis* spp., *Theileria* spp., *Babesia* spp., *Toxoplasma* spp.) were identified using both platforms, and five other genera (*Hepatozoon* spp., *Frenkelia* spp., *Isospora* spp., *Besnoitia* spp. *Eimeria* spp.) were identified with Nanopore sequencing only. At the species level, both Nanopore and Illumina platforms identified with more than 10 reads *Babesia* spp., *Sarcocystis neurona*, and *Theileria cervi*; moreover, the Nanopore platform identified with more than 10 reads *Toxoplasma gondii*, *Theileria* spp. *Theileria equi*, *Theileria ovis*, *Hepatozoon* spp., *Frenkelia glareoli*, *Sarcocystis dispersa*, and *Frenkelia microti*, while, the Illumina platform identified with more than one read, *Sarcocystis* spp., *Sarcocystis Scandinavica*, and *Sarcocystis miescheriana* (Table S1).

**Figure 3:**
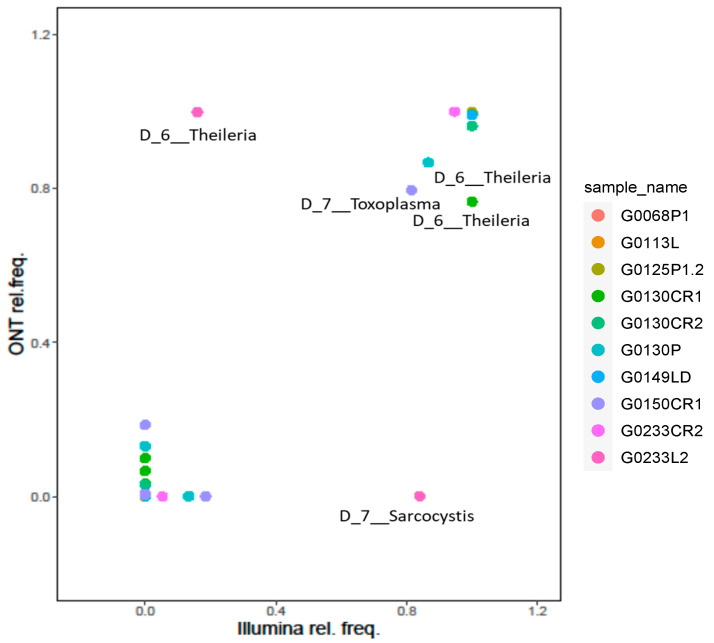
Nanopore and Illumina relative frequency at level 6. For each sample, the relative frequency of genera identified using Nanopore and Illumina platforms was reported, and those showing a marked difference between the two platforms were labelled with the genus name. Dots are coloured according to sample.

We then focused on the 10 samples classified as Apicomplexa positive by both platforms and evaluated their relative taxa abundance at level 6 (genus level). This resulted in a strong positive correlation (r Pearson = 0.86; *t*-test *p*-value = 2.6 × 10^−11^), confirming the soundness of the proposed approach (Figure 3).

## 4. Discussion

In this study, we describe Meat-Borne-Parasite, a Nanopore sequencing-based workflow for Apicomplexa detection in wildlife samples. According to the literature, this is the first meta-barcoding study on meat targeting protozoa. An innovative matrix was used to highlight the biodiversity of microorganisms, especially from an Amazonian Forest environment. Numerous research teams are increasingly using meta-barcoding for the biodiversity analysis of microbial ecology in various matrices (fish, faeces, soil, water). These innovative and powerful technologies are used in different fields for the detection of food frauds, identification of fish species or inspection of the dietary diversity of animals. However, bacteria are more frequently the target of the study, compared to parasites [19,31,32,33,34]. For example, Ludwig, A. et al., and Howells et al. described the presence of microorganisms such as parasites in meat, but it was not possible for them to establish potential co-carriage, due to the technologies used, hence the interest of our study [5,8].

Illumina MiSeq is the gold-standard and most frequently used platform in microbial ecology studies to describe biodiversity, but in recent years Oxford Nanopore Technologies has raised a lot of interest, thanks to the low price, portability, real-time features, and capability to sequence long reads on the MinION device. Despite many studies showing the reliability of Nanopore sequencing for a variety of applications [35,36], the higher error rate compared to Illumina (modal accuracy with R9.4.1 chemistry is at about 96%, Figure S1) requires ad hoc bioinformatic pipelines and thorough validation studies. Moreover, advancements both in the base-calling algorithms and in the sequencing chemistry have greatly improved Nanopore sequencing accuracy. The latest “Q20+” chemistry, which was recently released on the market, allows the production of sequencing reads with sequencing accuracy higher than 99% [37,38].

Multiple studies have already focused on comparing taxa abundances provided by Nanopore and Illumina-based workflows, showing a positive correlation, although some others showed poor correlation [39,40,41,42]. In general, multiple factors concur in determining taxa abundances, such as the reads sequencing accuracy, bioinformatic pipeline, reference database (accessed on 5 January 2023), and PCR primers used for amplification. In fact, due to an Illumina fragment length limited to about 600–800 bp, different PCR primer pairs are frequently used with the two platforms, greatly reducing the reciprocal overlap [43,44].

In this work, we validated our novel Meat-Borne-Parasite workflow, using Illumina platform as a gold-standard reference. In our workflow, the bioinformatic analysis is carried out using MetONTIIME, a novel QIIME2 pipeline based on Nextflow workflow manager, which exploits containerized technology for running all the steps consequentially in a resource-optimized way, enabling the streamlined analysis of multiple samples.

At the genus level, we obtained a Pearson correlation value of 0.86 between the two platforms. Overall, we find a good agreement between the two platforms at up to the genus level, further reinforcing the accuracy of the proposed approach. Conversely, a Pearson correlation value of 0.31 was obtained at the species level, suggesting caution should be used for species-level assignments with meta-barcoding workflows. Indeed, higher confidence in species-level assignments could be obtained using whole-genome sequencing or, at least, by combining information of multiple marker genes.

While the Illumina platform allowed the detection of the presence of Apicomplexa in 14/14 samples, our Meat-Borne-Parasite workflow allowed us to classify as Apicomplexa positive 10/14 samples, based on a conservative scoring rule which requires strong evidence of reads of Apicomplexa origin, to classify a sample as positive. This rule accounts for residual demultiplexing errors, which may occur due to sequencing errors in the barcode regions. The four discordant samples were characterized by low relative abundance in Illumina analysis, namely 0.22%, 0.09%, 0.04%, and 0.02%, respectively, in samples G0159P, G0173CR, G0173L, and G0225CR1. Therefore, the reasons why they were missed with Meat-Borne-Parasite workflow may be ascribed to a lower sequencing throughput (about 15% of Illumina reads were sequenced with Nanopore), or to differential PCR primers efficiency. The lower sensitivity issue should be largely mitigated, both increasing the sequencing run time and using the newest R10.4.1 chemistry, which will enable higher sequencing accuracy and, in turn, further reduce demultiplexing errors. Moreover, using smaller Flongle flow-cells for sequencing one sample at a time may represent an effective—although slightly more expensive—solution for higher sensitivity assays, by removing the need for demultiplexing.

Both platforms detected multiple co-occurring Apicomplexa genera, with a single species, *Dasyprocta leoprina*, carriing a single parasite, *Theileria* spp.; however, this can be explained by the fact that we only studied a single individual for this animal species, and more samples from this species should be analysed to have a better representation. Some parasites, such as *Toxoplasma gondii*, are known to show tropism: accordingly, we detected it only in the heart (one sample out of five). Conversely, *Babesia* spp. was found to be spread across all organs analysed in the present study. This could also be explained by the choice of analytical methods, in particular extraction, which could influence parasite detection. Indeed, *Toxoplasma gondii* requires specific isolation, as the parasite forms cysts in cell tissue, as described by Dubey et al. [45]. For this reason, other parasites, such as *Babesia* spp., are more frequently observed in various samples [46]. By exploiting our novel analysis workflow, a large-scale study will have to be carried out, taking into account analytical specificities as is the case for *Toxoplasma gondii*.

Finally, the Nanopore platform appears to be the most efficient one for global biodiversity parasites in wildlife species monitoring, and it can be used as a relevant tool for epidemiological or ecological surveillance on potentially human and animal pathogenic parasites, such as *Sarcocystis* spp., *Babesia* spp., and *Toxoplasma gondii* [3,12,47]. Further or complementary analyses using specific qPCR should be carried out to specifically determine the prevalence of species potentially pathogenic to animals and humans.

## 5. Conclusions

In this study, we showed the set-up of a novel workflow for Nanopore-based meta-barcoding aimed at detecting Apicomplexa infections in animal tissues. This protocol is simple and does not require extensive knowledge, such as morphological taxonomic identification skills. The strong correlation between the two sequencing platforms guarantees highly accurate genus-level results.

Moreover, this study shows a parasitic abundance in the wild fauna of French Guiana and allows the evaluation of several potential risks for humans and animals: the ecological risk, with predominant new or emerging parasitic strains, that could decimate animal species and the food risk for the population, since wild meat consumption is widespread. Thanks to low-cost, portability, and real-time sequencing, Nanopore-based meta-barcoding could enable local authorities to set up a surveillance system, thus contributing effectively to environmental biomonitoring tasks.

## Figures and Tables

**Table 1:** Distribution of host species studied.

Order	Family	Species	Common Name	Individual Distribution
**Rodentia**	Agoutidae	*Cuniculus paca*	Lowland paca, Paca	1
**Rodentia**	Dasyproctidae	*Dasyprocta leporina*	Red-rumped agouti	1
**Cingulata**	Dasypodidae	*Dasypus* sp. nov.	Nine-banded armadillo	7
**Rodentia**	Caviidae	*Hydrochoerus hydrochaeris*	Capybara	3
**Cetartiodactyla**	Cervidae	*Mazama americana*	Red brocket deer	2
**Perissodactyla**	Tapiridae	*Tapirus terrestris*	Tapir	1
			**Total**	**15**

**Table 2:** Demultiplexed sequence counts summary.

	Illumina Reads	Nanopore Reads
**Minimum**	71,565	18
**Median**	78,510	5821
**Mean**	78,495.5	11,127.4
**Maximum**	91,032	42,449
**Total**	1,569,910	233,676

**Table 3:** Host species corresponding to the identification codes.

Host Identification Code	Host Species
**G0068**	*Hydrochoerus hydrochaeris*
**G0113**	*Cuniculus paca*
**G0125**	*Mazama americana*
**G0130**	*Tapirus terrestris*
**G0149**	*Dasypus* sp. nov.
**G0173**	*Dasyprocta leporina*
**G0225**	*Dasypus* sp. nov.
**G0233**	*Mazama americana*

## Data Availability

Raw sequence reads generated in this study have been submitted to the SRA database (BioProject PRJNA1009124) (accessed on July 2023).

## Supplementary Materials

The following supporting information can be downloaded at https://www.mdpi.com/article/10.3390/cimb46050237/s1, Figure S1. Nanopore sequencing reads alignment identity distribution; Table S1. Nanopore and Illumina absolute frequency of Apicomplexa-assigned reads at taxonomic level 7.
