# Supplementary material for: Meat-Borne-Parasite: A Nanopore-Based Meta-Barcoding Work-Flow for Parasitic Microbiodiversity Assessment in the Wild Fauna of French Guiana
Source: Curr Issues Mol Biol. 2024 Apr 24;46(5):3810–21. doi: 10.3390/cimb46050237 (PMC11119736; doi:10.3390/cimb46050237)
Supplement: Supplementary file 1 [file cimb-46-00237-s001.zip › cimb-2890535-supplementary-Figure S1.pdf]

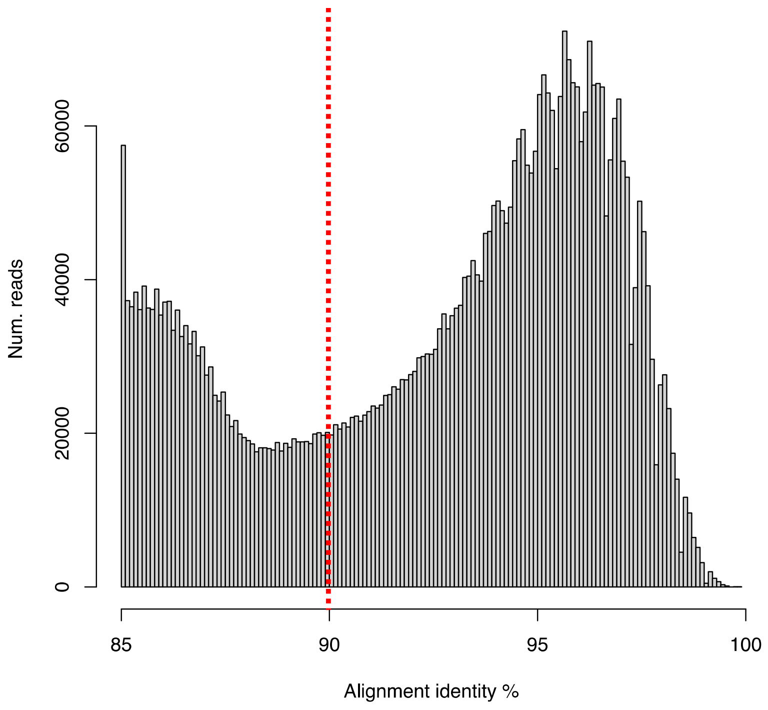

Figure S1. Nanopore sequencing reads alignment identity distribution. The alignment identity of Nanopore sequencing reads to Silva database is shown. A dashed red line represents minimum identity threshold used for filtering hits.
