# Supplementary material for: Meat-Borne-Parasite: A Nanopore-Based Meta-Barcoding Work-Flow for Parasitic Microbiodiversity Assessment in the Wild Fauna of French Guiana
Source: Curr Issues Mol Biol. 2024 Apr 24;46(5):3810–21. doi: 10.3390/cimb46050237 (PMC11119736; doi:10.3390/cimb46050237)
Supplement: Supplementary file 1 [file cimb-46-00237-s001.zip › cimb-2890535-supplementary-Table S1.pdf]

Table S1. Nanopore and Illumina absolute frequency of Apicomplexa-assigned reads at taxonomic level 7.

Constructed from biom file
Animal Species
Tissues
#OTU ID
D_0__Eukaryota;D_1__SAR;D_2__Alveolata;D_3__Apicomplexa;D_5__Piroplasmorida;D_6__Theileria;D_7__Theileria cervi
D_0__Eukaryota;D_1__SAR;D_2__Alveolata;D_3__Apicomplexa;D_6__Eimeriorina;D_7__Sarcocystis;__
D_0__Eukaryota;D_1__SAR;D_2__Alveolata;D_3__Apicomplexa;D_5__Piroplasmorida;D_6__Theileria;D_7__Theileria sp. NG-2012b
D_0__Eukaryota;D_1__SAR;D_2__Alveolata;D_3__Apicomplexa;D_5__Coccidia;D_6__Eimeriorina;__
D_0__Eukaryota;D_1__SAR;D_2__Alveolata;D_3__Apicomplexa;D_6__Eimeriorina;D_7__Toxoplasma;__
D_0__Eukaryota;D_1__SAR;D_2__Alveolata;D_3__Apicomplexa;D_6__Eimeriorina;D_7__Sarcocystis;D_8__Sarcocystis neurona
D_0__Eukaryota;D_1__SAR;D_2__Alveolata;D_3__Apicomplexa;D_5__Piroplasmorida;D_6__Babesia;D_7__Babesia sp. capybara 1
D_0__Eukaryota;D_1__SAR;D_2__Alveolata;D_3__Apicomplexa;D_5__Piroplasmorida;D_6__Theileria;D_7__Theileria sp. 2 PJH-2012
D_0__Eukaryota;D_1__SAR;D_2__Alveolata;D_3__Apicomplexa;D_5__Piroplasmorida;D_6__Theileria;D_7__Theileria equi
D_0__Eukaryota;D_1__SAR;D_2__Alveolata;D_3__Apicomplexa;D_5__Piroplasmorida;D_6__Theileria;__
D_0__Eukaryota;D_1__SAR;D_2__Alveolata;D_3__Apicomplexa;D_5__Piroplasmorida;D_6__Theileria;D_7__Theileria sp. NG-2013a
D_0__Eukaryota;D_1__SAR;D_2__Alveolata;D_3__Apicomplexa;D_6__Eimeriorina;__;__
D_0__Eukaryota;D_1__SAR;D_2__Alveolata;D_3__Apicomplexa;D_5__Coccidia;D_6__Eimeriorina;D_7__Coccidia sp. ex Cricetus cricetus
D_0__Eukaryota;D_1__SAR;D_2__Alveolata;D_3__Apicomplexa;__;__;__
D_0__Eukaryota;D_1__SAR;D_2__Alveolata;D_3__Apicomplexa;D_5__Coccidia;D_6__Eimeriorina;D_7__Eimeriidae sp. RY-2014
D_0__Eukaryota;D_1__SAR;D_2__Alveolata;D_3__Apicomplexa;D_5__Piroplasmorida;D_6__Theileria;D_7__Theileria ovis
D_0__Eukaryota;D_1__SAR;D_2__Alveolata;D_3__Apicomplexa;D_5__Piroplasmorida;D_6__Theileria;D_7__Theileria equi strain WA
D_0__Eukaryota;D_1__SAR;D_2__Alveolata;D_3__Apicomplexa;D_5__Piroplasmorida;D_6__Theileria;D_7__Theileria sp.
D_0__Eukaryota;D_1__SAR;D_2__Alveolata;D_3__Apicomplexa;D_6__Adeleorina;D_7__Hepatozoon;__
D_0__Eukaryota;D_1__SAR;D_2__Alveolata;D_3__Apicomplexa;D_6__Adeleorina;D_7__Hepatozoon;D_8__Hepatozoon sp. European pine marten 1
D_0__Eukaryota;D_1__SAR;D_2__Alveolata;D_3__Apicomplexa;D_6__Eimeriorina;D_7__Sarcocystis;D_8__Frenkelia glareoli
D_0__Eukaryota;D_1__SAR;D_2__Alveolata;D_3__Apicomplexa;D_6__Eimeriorina;D_7__Besnoitia;D_8__Besnoitia bennetti
D_0__Eukaryota;D_1__SAR;D_2__Alveolata;D_3__Apicomplexa;D_6__Adeleorina;D_7__Hepatozoon;D_8__Hepatozoon sp. I35
D_0__Eukaryota;D_1__SAR;D_2__Alveolata;D_3__Apicomplexa;D_5__Piroplasmorida;D_6__Theileria;D_7__Theileria sp. G164
D_0__Eukaryota;D_1__SAR;D_2__Alveolata;D_3__Apicomplexa;D_6__Eimeriorina;D_7__Sarcocystis;D_8__Sarcocystis dispersa
D_0__Eukaryota;D_1__SAR;D_2__Alveolata;D_3__Apicomplexa;D_6__Eimeriorina;D_7__Frenkelia;D_8__Frenkelia microti
D_0__Eukaryota;D_1__SAR;D_2__Alveolata;D_3__Apicomplexa;D_6__Eimeriorina;D_7__Sarcocystis;D_8__Sarcocystis miescheriana
D_0__Eukaryota;D_1__SAR;D_2__Alveolata;D_3__Apicomplexa;D_6__Eimeriorina;D_7__Sarcocystis;D_8__Sarcocystis sp.
D_0__Eukaryota;D_1__SAR;D_2__Alveolata;D_3__Apicomplexa;D_6__Eimeriorina;D_7__Sarcocystis;D_8__Sarcocystis scandinavica
D_0__Eukaryota;D_1__SAR;D_2__Alveolata;D_3__Apicomplexa;D_5__Piroplasmorida;D_6__Theileria;D_7__Theileria sp. North Texas white-tailed deer D_0__Eukaryota;D_1__SAR;D_2__Alveolata;D_3__Apicomplexa;D_6__Eimeriorina;D_7__Toxoplasma;D_8__Ovis aries musimon (mouflon)
D_0__Eukaryota;D_1__SAR;D_2__Alveolata;D_3__Apicomplexa;D_5__Piroplasmorida;D_6__Theileria;D_7__Theileria sp. NG-2013c
D_0__Eukaryota;D_1__SAR;D_2__Alveolata;D_3__Apicomplexa;D_6__Eimeriorina;D_7__Isospora;__
D_0__Eukaryota;D_1__SAR;D_2__Alveolata;D_3__Apicomplexa;D_5__Piroplasmorida;D_6__Babesia;__
D_0__Eukaryota;D_1__SAR;D_2__Alveolata;D_3__Apicomplexa;D_5__Piroplasmorida;D_6__Babesia;D_7__Babesia sp. MA#230
D_0__Eukaryota;D_1__SAR;D_2__Alveolata;D_3__Apicomplexa;D_6__Eimeriorina;D_7__Isospora;D_8__Isospora wiegmanniana
D_0__Eukaryota;D_1__SAR;D_2__Alveolata;D_3__Apicomplexa;D_6__Eimeriorina;D_7__Besnoitia;__
D_0__Eukaryota;D_1__SAR;D_2__Alveolata;D_3__Apicomplexa;D_5__Piroplasmorida;D_6__Babesia;D_7__Babesia sp. sable antelope/2005
D_0__Eukaryota;D_1__SAR;D_2__Alveolata;D_3__Apicomplexa;D_5__Piroplasmorida;D_6__Theileria;D_7__Theileria sp. ex Syncerus caffer MCO-2011 D_0__Eukaryota;D_1__SAR;D_2__Alveolata;D_3__Apicomplexa;D_6__Eimeriorina;D_7__Sarcocystis;D_8__Sarcocystis arctica
D_0__Eukaryota;D_1__SAR;D_2__Alveolata;D_3__Apicomplexa;D_6__Eugregarinorida;__;__
D_0__Eukaryota;D_1__SAR;D_2__Alveolata;D_3__Apicomplexa;D_5__Piroplasmorida;D_6__Babesia;D_7__Babesia sp. 1-1658
D_0__Eukaryota;D_1__SAR;D_2__Alveolata;D_3__Apicomplexa;D_6__Eimeriorina;D_7__Toxoplasma;D_8__Toxoplasma gondii RH
D_0__Eukaryota;D_1__SAR;D_2__Alveolata;D_3__Apicomplexa;D_6__Adeleorina;D_7__Hepatozoon;D_8__Hepatozoon ursi
D_0__Eukaryota;D_1__SAR;D_2__Alveolata;D_3__Apicomplexa;D_6__Eimeriorina;D_7__Eimeria;__
D_0__Eukaryota;D_1__SAR;D_2__Alveolata;D_3__Apicomplexa;D_5__Piroplasmorida;D_6__Theileria;D_7__Theileria sp. H8

Hydrochoerus hydrochaeris	Cuniculus paca	Mazama americana	Tapirus terrestris	Tapirus terrestris	Tapirus terrestris
Lung	Tongue	Lung	Heart	Heart	Lung
G0068P1_Illumina	G0113L_Illumina	G0125P1.2_Illumina	G0130CR1_Illumina	G0130CR2_Illumina	G0130P_Illumina
0.0	0.0	289.0	0.0	0.0	0.0
0.0	0.0	0.0	0.0	0.0	0.0
0.0	0.0	0.0	0.0	0.0	0.0
0.0	0.0	0.0	0.0	0.0	0.0
0.0	0.0	0.0	0.0	0.0	0.0
0.0	773.0	0.0	0.0	0.0	0.0
17.0	0.0	0.0	0.0	0.0	0.0
0.0	0.0	0.0	0.0	0.0	0.0
0.0	0.0	0.0	0.0	0.0	0.0
0.0	0.0	8.0	122.0	28.0	13.0
0.0	0.0	0.0	0.0	0.0	0.0
0.0	0.0	0.0	0.0	0.0	0.0
0.0	0.0	0.0	0.0	0.0	0.0
0.0	0.0	0.0	0.0	0.0	0.0
0.0	0.0	0.0	0.0	0.0	0.0
0.0	0.0	0.0	0.0	0.0	0.0
0.0	0.0	0.0	0.0	0.0	0.0
0.0	0.0	0.0	0.0	0.0	0.0
0.0	0.0	0.0	0.0	0.0	0.0
0.0	0.0	0.0	0.0	0.0	0.0
0.0	0.0	0.0	0.0	0.0	0.0
0.0	0.0	0.0	0.0	0.0	0.0
0.0	0.0	0.0	0.0	0.0	0.0
0.0	0.0	0.0	0.0	0.0	0.0
0.0	0.0	0.0	0.0	0.0	0.0
0.0	0.0	0.0	0.0	0.0	0.0
0.0	0.0	0.0	0.0	0.0	0.0
0.0	0.0	0.0	0.0	0.0	0.0
0.0	0.0	0.0	0.0	0.0	0.0
0.0	0.0	0.0	0.0	0.0	0.0
0.0	0.0	0.0	0.0	0.0	0.0
0.0	0.0	0.0	0.0	0.0	0.0
0.0	0.0	0.0	0.0	0.0	0.0
0.0	0.0	0.0	0.0	0.0	2.0
0.0	0.0	0.0	0.0	0.0	0.0
0.0	0.0	0.0	0.0	0.0	0.0
0.0	0.0	0.0	0.0	0.0	0.0
0.0	0.0	0.0	0.0	0.0	0.0
0.0	0.0	0.0	0.0	0.0	0.0
0.0	0.0	0.0	0.0	0.0	0.0
0.0	0.0	0.0	0.0	0.0	0.0
0.0	0.0	0.0	0.0	0.0	0.0
0.0	0.0	0.0	0.0	0.0	0.0
0.0	0.0	0.0	0.0	0.0	0.0
0.0	0.0	0.0	0.0	0.0	0.0

Dasypus sp. nov.<br>Tongue<br>G0149LD_Illumina	Cuniculus paca<br>Heart<br>G0150CR1_Illumina	Dasypus sp. nov.<br>Lung<br>G0159P_Illumina	Dasyprocta leporina<br>Lung<br>G0173L_Illumina	Dasyprocta leporina<br>Heart<br>G0173CR_Illumina	Dasypus sp. nov.<br>Heart<br>G0225CR1_Illumina
0.0	0.0	0.0	0.0	0.0	0.0
34.0	5.0	0.0	0.0	0.0	0.0
0.0	0.0	0.0	0.0	0.0	0.0
0.0	0.0	0.0	0.0	0.0	0.0
0.0	22.0	0.0	0.0	0.0	0.0
264.0	0.0	0.0	0.0	0.0	0.0
0.0	0.0	0.0	0.0	0.0	0.0
0.0	0.0	0.0	0.0	0.0	0.0
0.0	0.0	0.0	0.0	0.0	0.0
0.0	0.0	0.0	3.0	72.0	0.0
0.0	0.0	0.0	0.0	0.0	0.0
0.0	0.0	0.0	0.0	0.0	0.0
0.0	0.0	0.0	0.0	0.0	0.0
0.0	0.0	185.0	0.0	0.0	17.0
0.0	0.0	0.0	0.0	0.0	0.0
0.0	0.0	0.0	0.0	0.0	0.0
0.0	0.0	0.0	0.0	0.0	0.0
0.0	0.0	0.0	0.0	0.0	0.0
0.0	0.0	0.0	0.0	0.0	0.0
0.0	0.0	0.0	0.0	0.0	0.0
0.0	0.0	0.0	0.0	0.0	0.0
0.0	0.0	0.0	0.0	0.0	0.0
0.0	0.0	0.0	0.0	0.0	0.0
0.0	0.0	0.0	0.0	0.0	0.0
0.0	0.0	0.0	0.0	0.0	0.0
0.0	0.0	0.0	0.0	0.0	0.0
84.0	0.0	0.0	0.0	0.0	0.0
0.0	0.0	0.0	0.0	0.0	0.0
0.0	0.0	0.0	0.0	0.0	0.0
0.0	0.0	0.0	0.0	0.0	0.0
0.0	0.0	0.0	0.0	0.0	0.0
0.0	0.0	0.0	0.0	0.0	0.0
0.0	0.0	0.0	0.0	0.0	0.0
0.0	0.0	0.0	0.0	0.0	0.0
0.0	0.0	0.0	0.0	0.0	0.0
0.0	0.0	0.0	0.0	0.0	0.0
0.0	0.0	0.0	0.0	0.0	0.0
0.0	0.0	0.0	0.0	0.0	0.0
0.0	0.0	0.0	0.0	0.0	0.0
0.0	0.0	0.0	0.0	0.0	0.0
0.0	0.0	17.0	0.0	0.0	0.0
0.0	0.0	0.0	0.0	0.0	0.0
0.0	0.0	0.0	0.0	0.0	0.0
0.0	0.0	0.0	0.0	0.0	0.0
0.0	0.0	0.0	0.0	0.0	0.0
0.0	0.0	0.0	0.0	0.0	0.0

Mazama americana Tingue G0233L2_Illumina	Mazama americana Heart G0233CR2_Illumina	Hydrochoerus hydrochaeris Lung G0068P1_ONT	Cuniculus paca Tongue G0113L_ONT	Mazama americana Lung G0125P1.2_ONT	Tapirus terrestris Heart G0130CR1_ONT
75.0	637.0	1.0	1.0	4448.0	0.0
506.0	98.0	0.0	19766.0	1.0	0.0
0.0	0.0	0.0	0.0	3127.0	0.0
0.0	0.0	0.0	0.0	0.0	0.0
0.0	0.0	0.0	0.0	0.0	0.0
0.0	0.0	0.0	1029.0	0.0	0.0
0.0	0.0	6280.0	1.0	0.0	0.0
0.0	0.0	0.0	0.0	451.0	0.0
0.0	0.0	0.0	0.0	0.0	18.0
67.0	1098.0	0.0	0.0	397.0	2.0
0.0	0.0	0.0	0.0	0.0	3.0
0.0	0.0	0.0	96.0	0.0	2.0
0.0	0.0	0.0	0.0	0.0	0.0
0.0	0.0	0.0	0.0	0.0	1.0
0.0	0.0	0.0	0.0	0.0	0.0
0.0	0.0	0.0	0.0	82.0	0.0
0.0	0.0	0.0	0.0	0.0	0.0
0.0	0.0	0.0	0.0	0.0	0.0
0.0	0.0	0.0	0.0	0.0	0.0
0.0	0.0	0.0	0.0	0.0	0.0
0.0	0.0	0.0	66.0	0.0	0.0
0.0	0.0	0.0	0.0	0.0	0.0
0.0	0.0	0.0	0.0	0.0	0.0
0.0	0.0	0.0	0.0	22.0	0.0
0.0	0.0	0.0	14.0	0.0	0.0
0.0	0.0	0.0	15.0	0.0	0.0
0.0	0.0	0.0	0.0	0.0	0.0
0.0	0.0	0.0	65.0	0.0	0.0
241.0	0.0	0.0	0.0	0.0	0.0
0.0	0.0	0.0	0.0	5.0	0.0
0.0	0.0	0.0	0.0	0.0	0.0
0.0	0.0	0.0	0.0	6.0	0.0
0.0	0.0	0.0	0.0	0.0	2.0
0.0	0.0	0.0	0.0	0.0	0.0
0.0	0.0	0.0	0.0	0.0	0.0
0.0	0.0	0.0	0.0	0.0	1.0
0.0	0.0	0.0	0.0	0.0	0.0
0.0	0.0	0.0	0.0	0.0	0.0
0.0	0.0	0.0	0.0	1.0	0.0
0.0	0.0	0.0	1.0	0.0	0.0
0.0	0.0	0.0	0.0	0.0	0.0
0.0	0.0	0.0	0.0	0.0	0.0
0.0	0.0	0.0	0.0	0.0	0.0
0.0	0.0	0.0	0.0	0.0	0.0
0.0	0.0	0.0	0.0	0.0	1.0
0.0	0.0	0.0	0.0	0.0	0.0

Tapirus terrestris<br>Heart<br>G0130CR2_ONT	Tapirus terrestris<br>Lung<br>G0130P_ONT	Dasypus sp. nov.<br>Tongue<br>G0149LD_ONT	Cuniculus paca<br>Heart<br>G0150CR1_ONT	Mazama americana<br>Tongue<br>G0233L2_ONT	Mazama americana<br>Heart<br>G0233CR2_ONT
0.0	1.0	0.0	0.0	2239.0	3107.0
0.0	1.0	3118.0	0.0	2.0	0.0
0.0	0.0	0.0	0.0	939.0	493.0
0.0	0.0	0.0	143.0	0.0	0.0
0.0	0.0	0.0	643.0	0.0	0.0
0.0	0.0	173.0	0.0	0.0	0.0
0.0	0.0	0.0	0.0	0.0	0.0
0.0	0.0	0.0	0.0	694.0	592.0
261.0	742.0	0.0	0.0	0.0	0.0
37.0	72.0	0.0	0.0	776.0	1230.0
31.0	127.0	0.0	0.0	0.0	0.0
0.0	0.0	21.0	7.0	0.0	0.0
0.0	0.0	0.0	2.0	0.0	0.0
0.0	0.0	0.0	3.0	0.0	0.0
0.0	0.0	0.0	6.0	0.0	0.0
0.0	0.0	0.0	0.0	53.0	29.0
11.0	87.0	0.0	0.0	0.0	0.0
0.0	0.0	0.0	0.0	1.0	0.0
0.0	122.0	0.0	0.0	0.0	0.0
0.0	15.0	0.0	0.0	0.0	0.0
0.0	0.0	23.0	0.0	0.0	0.0
0.0	0.0	0.0	4.0	0.0	0.0
0.0	17.0	0.0	0.0	0.0	0.0
0.0	0.0	0.0	0.0	8.0	3.0
0.0	0.0	0.0	0.0	0.0	0.0
0.0	0.0	9.0	0.0	0.0	0.0
0.0	0.0	4.0	0.0	0.0	0.0
0.0	0.0	7.0	0.0	0.0	0.0
0.0	0.0	0.0	0.0	4.0	0.0
0.0	0.0	0.0	0.0	13.0	23.0
0.0	0.0	0.0	1.0	0.0	0.0
0.0	0.0	0.0	0.0	1.0	0.0
2.0	0.0	0.0	0.0	0.0	0.0
8.0	1.0	0.0	0.0	0.0	0.0
1.0	0.0	0.0	0.0	0.0	0.0
0.0	0.0	0.0	0.0	0.0	0.0
0.0	0.0	0.0	0.0	0.0	0.0
1.0	0.0	0.0	0.0	0.0	0.0
0.0	0.0	0.0	0.0	0.0	0.0
0.0	0.0	0.0	0.0	0.0	0.0
0.0	0.0	0.0	0.0	0.0	0.0
1.0	0.0	0.0	0.0	0.0	0.0
0.0	0.0	0.0	0.0	0.0	0.0
0.0	1.0	0.0	0.0	0.0	0.0
0.0	0.0	0.0	0.0	0.0	0.0
0.0	0.0	0.0	0.0	1.0	0.0
